# Two Newly Identified *Colletotrichum* Species Associated with Mango Anthracnose in Central Thailand

**DOI:** 10.3390/plants12051130

**Published:** 2023-03-02

**Authors:** Chainarong Rattanakreetakul, Pisut Keawmanee, Santiti Bincader, Orarat Mongkolporn, Vipaporn Phuntumart, Sotaro Chiba, Ratiya Pongpisutta

**Affiliations:** 1 Department of Plant Pathology, Faculty of Agriculture at Kamphaeng Saen, Kasetsart University, Nakhon Pathom 73140, Thailand; 2 Program Plant Science, Agricultural Technology and Agro-Industry Faculty, Rajamangala University of Technology Suvarnabhumi, Phra Nakhon Si Ayutthaya 13000, Thailand; 3 Department of Horticulture, Faculty of Agriculture at Kamphaeng Saen, Kasetsart University, Nakhon Pathom 73140, Thailand; 4 Department of Biological Sciences, 129 Life Sciences Building, Bowling Green State University, Bowling Green, OH 43403, USA; 5 Graduate School of Bioagricultural Sciences, Nagoya University, Nagoya 464-8601, Japan

**Keywords:** *Colletotrichum*, *Mangifera indica* L., species identification, multilocus phylogeny

## Abstract

Anthracnose caused by *Colletotrichum* spp. is one of the major problems in mango production worldwide, including Thailand. All mango cultivars are susceptible, but Nam Dok Mai See Thong (NDMST) is the most vulnerable. Through a single spore isolation method, a total of 37 isolates of *Colletotrichum* spp. were obtained from NDMST showing anthracnose symptoms. Identification was performed using a combination of morphology characteristics, Koch’s postulates, and phylogenetic analysis. The pathogenicity assay and Koch’s postulates on leaves and fruit confirmed that all *Colletotrichum* spp. tested were causal agents of mango anthracnose. Multilocus analysis using DNA sequences of internal transcribed spacer (ITS) regions, β-tubulin (*TUB2*), actin (*ACT*), and chitin synthase (*CHS-1*) was performed for molecular identification. Two concatenated phylogenetic trees were constructed using either two-loci of ITS and *TUB2*, or four-loci of ITS, *TUB2*, *ACT*, and *CHS-1.* Both phylogenetic trees were indistinguishable and showed that these 37 isolates belong to *C. acutatum*, *C. asianum*, *C. gloeosporioides*, and *C. siamense.* Our results indicated that using at least two loci of ITS and *TUB2,* were sufficient to infer *Colletotrichum* species complexes. Of 37 isolates, *C. gloeosporioides* was the most dominant species (19 isolates), followed by *C. asianum* (10 isolates), *C. acutatum* (5 isolates), and *C. siamense* (3 isolates). In Thailand, *C. gloeosporioides* and *C. acutatum* have been reported to cause anthracnose in mango, however, this is the first report of *C. asianum* and *C. siamense* associated with mango anthracnose in central Thailand.

## 1. Introduction

Mango production has expanded to more than 100 countries, with around 44.6 M tons annually since 2018 [1]. Thailand is a major producer and contributes to almost 8% of the global mango production [1]. Because of its flavor and texture, Nam Dok Mai See Thong (NDMST) has become the most popular mango cultivar in Thailand. It is also an early-midseason cultivar, which means that it has the potential to produce fruit all year round [2,3]. Anthracnose caused by *Colletotrichum* spp. is a significant economic problem in both mango orchards and postharvest storage [3,4,5,6,7,8]. The pathogen infects not only fruit but also inflorescences, flowers, and leaves. During the flowering stage, especially under high humidity, a disease incidence of 100% has been observed [4,9,10,11,12]. Young leaves that emerge during rainy periods are also prone to anthracnose infection. Anthracnose symptoms on leaves appear as small and dark brown spots often surrounded with chlorotic haloes and irregular margins [2,6,9,12,13]. Leaf lesions usually remain small, however, under disease-favorable conditions, the lesions can enlarge and fuse together to form irregular patches [2,4,5,14]. Other symptoms include premature leaf drop and twig dieback [4,15,16]. Mango fruit at any stage can be infected [5,17,18,19,20]. Mummification was usually observed in young fruit, while no symptom was observed in mature unripe fruit. On ripe fruit, dark brown irregular lesions appear, which gradually increase in irregular size and under favorable conditions, salmon to orange fungal conidial masses can be observed on the lesions [4,5,14,21]. The application of fungicides is a common practice to control anthracnose in mango orchards in Thailand, although it is often unsuccessful. The inefficient fungicide application is probably due to the emergence of new *Colletotrichum* species and/or fungicide resistance of the pathogens.

Accurate identification of species is a necessary starting point for the effective management of anthracnose disease. Currently, three *Colletotrichum* species; *C. acutatum*, *C. boninense*, and *C. gloeosporioides* have been reported to cause anthracnose in mango [22,23,24]. However, *C. gloeosporioides* species complex, which are ubiquitous fungal pathogens in tropical and sub-tropical areas, has not been reported in Thailand. The host-association and morphological characteristics that have been used to identify *Colletotrichum* species [25,26] are insufficient to distinguish these pathogens at the species level due to the limited number of morphological characters and the pathogens are not host specific. A polyphasic approach, multilocus phylogenetic analysis in conjunction with recognizable phenotypic characters, has been recommended to accurately identify species within the *Colletotrichum* genus [27,28]. According to Weir et al. [27], internal transcribed spacer (ITS) sequences alone are not reliable to distinguish different species within the *C. gloeosporioides* species complex. Additional recommended loci for the identification of the *Colletotrichum* species included actin (*ACT*), calmodulin (*CAL*), chitin synthase-1 (*CHS-1*), glyceraldehyde-3-phosphate dehydrogenase (*GAPDH*), and β-tubulin (*TUB2*) [7,9,23,26,28,29,30]. The identification of *C. gloeosporioides* species complex has not been fully investigated in mango anthracnose in Thailand. The identification of *C. gloeosporioides* species complex has not been fully investigated in mango anthracnose in Thailand. We hypothesize that the *C. gloeosporioides* species complex could be a potential source of new *Colletotrichum* species. This study used an integrative approach of a morphological assay, multilocus phylogenetic analysis, and pathogenicity test to identify species of the *Colletotrichum* infecting mango NDMST in Thailand. Here, we report for the first time that *C. asianum* and *C. siamense* are casual fungi causing anthracnose in mango grown in central Thailand.

## 2. Results

### 2.1. Fungal Isolates

**Figure 1:**
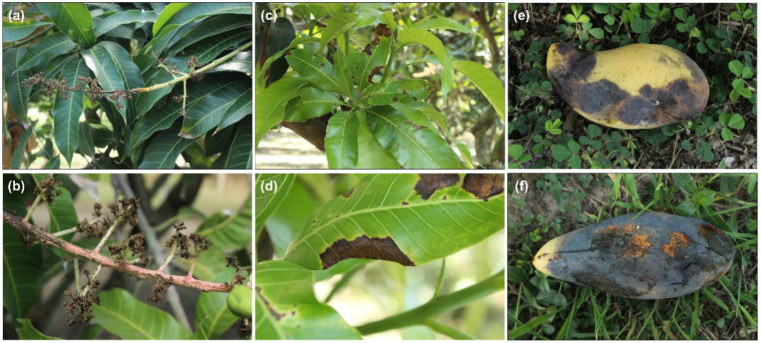
Visual symptoms of anthracnose caused by *Colletotrichum* spp. on mango tissues of NDMST cultivar; blight on inflorescence (**a**,**b**), irregular necrotic lesions on leaves (**c**,**d**) and irregular and sunken necrotic lesion on fruit (**e**) with abundant spore masses (**f**).

Inflorescence, leaves, and fruit showing typical anthracnose symptoms were collected from orchards located in Chachoengsao, Phichit and Ratchaburi. Visual anthracnose symptoms were inflorescence blight (Figure 1a,b), small brown to black spots and irregularly shaped lesions with brown to black necrotic lesions on leaves (Figure 1c,d). Symptoms on ripe fruit were small black circular spots, irregular and necrotic sunken brown to black lesions. On severely infected fruit, extensive fruit rot was observed (Figure 1e), under high humidity, bright orange to pale colored spore masses can be seen (Figure 1f). Infected premature fruit were usually dropped from the trees. A total of 37 fungal isolates resembling *Colletotrichum* spp. were obtained (Table 1).

### 2.2. Morphology-Based Identification

**Figure 2:**
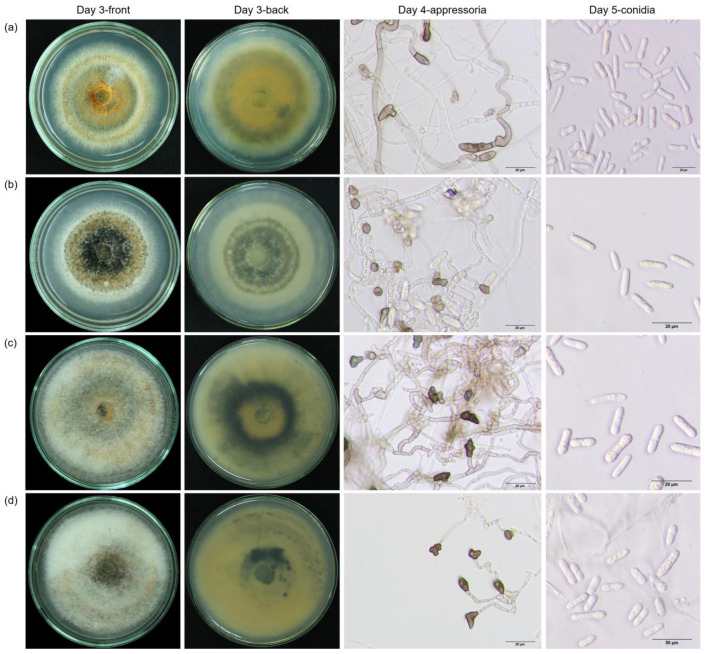
Morphological characteristics of colonies grown on PDA at day 3, pictures were taken from the front and the back of the plates. Appressoria were measured at day 4 and conidia were measured at day 5 after incubation. Shown here are the representatives of each species. *C. acutatum* PC011 (**a**), *C. asianum* RB001 (**b**), *C. gloeosporioides* CS005 (**c**), and *C. siamense* RB003 (**d**).

Based on colony morphology and conidia characteristics, the *Colletotrichum* isolates were classified into two groups. The first group contained five isolates that were similar to *C. acutatum*. The colony grown on PDA appeared white to grey and the reverse side of the colony was pale ochreous. The mycelial growth rates at day 3 ranged from 3.33–3.77 mm day^−1^ (average = 3.56 mm day^−1^). Spore masses were bright orange, conidia were hyaline, aseptate, straight, apex obtuse, and no setae. The sizes of conidia ranged from 3.38–6.17 µm (average = 4.43 µm) in width and from 10.96–19.88 µm (average = 14.8 µm) in length. Appressoria were clavate, long and irregular shapes, pale to dark brown in color. The diameter of appressoria ranged from 3.86–6.95 (average = 5.45 µm) in width and ranged from 5.97–11.20(8.36) µm (Table S1, Figure 2).

The second group contained 32 isolates with similarity to *C. gloeosporioides* (some of these isolates were later classified as *C. asianum* or *C. siamense* when DNA markers were integrated, described below). The colony grown on PDA showed a great variation in color from white, greenish to grayish, pale yellowish to dark grey, while the reverse sides were dark green (Figure 2). The mycelial growth rates also varied from 2.13 to 5.43 mm (average = 4.01 mm day^−1^). Conidia were hyaline, aseptate, straight cylindrical, rounded at the apex end and conspicuous hilum at basal end, and no setae. The sizes of conidia ranged from 3.60–7.07 µm (average = 5.03 µm) in width and ranged from 10.33–19.95 µm (average = 14.24 µm) in length (Table S1, Figure 2). Spore masses were pale, salmon-orange to bright orange colors. Various shapes of appressoria of clavate, long clavate, occasionally irregular, pale to dark brown in color were observed (Table S1, Figure 2). The size of the appressoria ranged from 4.42–8.09 µm (average = 5.82 µm) × 5.53–11.59 µm (average = 8.72 µm) (Table S1, Figure 2).

### 2.3. DNA Marker-Based Identification

**Figure 3:**
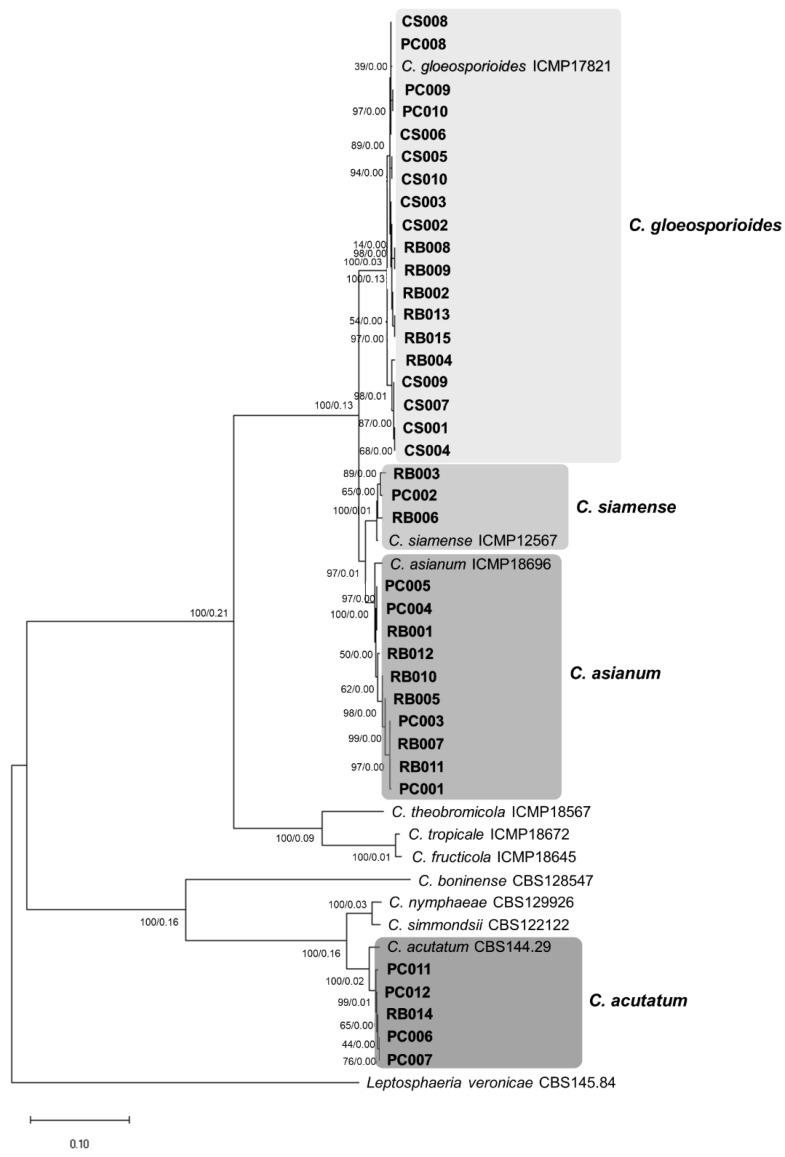
The maximum likelihood tree based on a concatenated data set of ITS and *TUB2* sequences of 47 *Colletotrichum* isolates, ex-type/epitype, were retrieved from GeneBank. Numbers on the node are bootstrap values (left) and posterior probability (right). *Colletotrichum* isolates from this study are in grey boxes. The scale bar shows the number of substitutions per site.

All DNA sequences of *ACT*, *CHS-1*, *ITS*, and *TUB2* were subjected to BLASTn (Table S2). Sequences of ex-type or epitype strains of *Colletotrichum* species (Table S2) were selected for phylogenetic analysis. We firstly constructed a phylogenetic tree using two DNA markers: ITS and *TUB2*. A total of 47 isolates, including ten isolates of ex-type and ex-epitype or epitype strains, and *Leptosphaeria veronicae* CBS145.84 was used as an outgroup (Figure 3). The topology of the ML tree and Bayesian tree were identical, therefore, only the ML tree is shown. A discrete Gamma distribution was used to estimate the divergence and evolutionary rate (+G, parameter = 7.1107) (Figure 3). The 37 isolates were assigned to four species clades on the maximum likelihood (ML) tree. Of these, 19 isolates formed a clade that was closely related to *C. gloeosporioides* strain (ICMP17821), showing 0.13 posterior probability with a bootstrap value of 100%, three isolates were clustered with *C. siamense* (ICMP12567) with 0.01 posterior probability and a bootstrap value of 100%; ten isolates clustered with *C. asianum* (ICMP18696) with 0.00 posterior probability and a bootstrap value of 97%, and five isolates were grouped with *C. acutatum* (CBS144.29) with 0.02 posterior probability and a bootstrap value of 100%.

**Figure 4:**
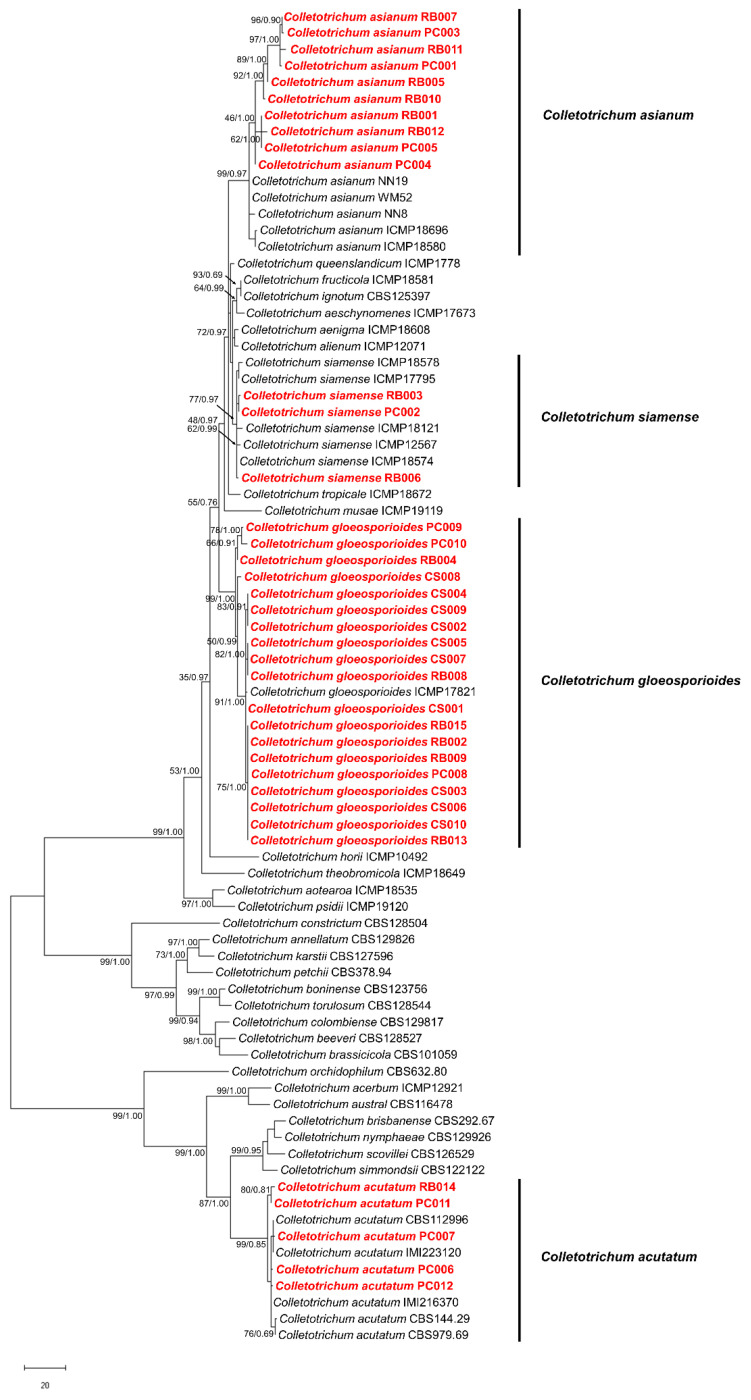
The maximum likelihood tree based on the concatenated data set of *ACT*, *CHS-1*, ITS, and *TUB2* sequences from a total of 81 *Colletotrichum* species with the ex-type/epitype were retrieved from GeneBank. Numbers on the node are bootstrap values (left) and posterior probability (right). *Colletotrichum* isolates from this study are in red. The scale bar shows the number of substitutions per site.

The incongruence length difference (ILD) test showed that the *ACT*, *CHS-1*, ITS, and *TUB2* were homogeneous. Therefore, the concatenated sequences of four markers; *ACT*, *CHS-1*, ITS, and *TUB2* were used to generate a phylogenetic tree with a total of 81 isolates, including 44 isolates of ex-type and ex-epitype or epitype strains. The topology of the ML tree was consistent with that of the Bayesian tree, and therefore only the ML tree is shown (Figure 4). Among the 37 isolates, ten isolates were clustered with *C. asianum* strains (ICMP 18580, ICMP 18696, NN8, WM52, and NN19) showing 0.97 posterior probability and with bootstrap values of 99%; three isolates formed a clade with *C. siamense* strains (ICMP18578, ICMP17795, ICMP18121, ICMP12567, and ICMP18574) with 0.97 posterior probability with bootstrap values of 77%; and 19 isolates clustered with the *C. gloeosporioides* strain (ICMP17821) with 1.00 posterior probability with bootstrap values of 99%. Five isolates were clustered with *C. acutatum* (CBS112996, IMI223120, IMI216370, CBS144.29, and CBS979.69) with 0.85 posterior probability with bootstrap values of 99%.

### 2.4. Pathogenicity Test

**Figure 5:**
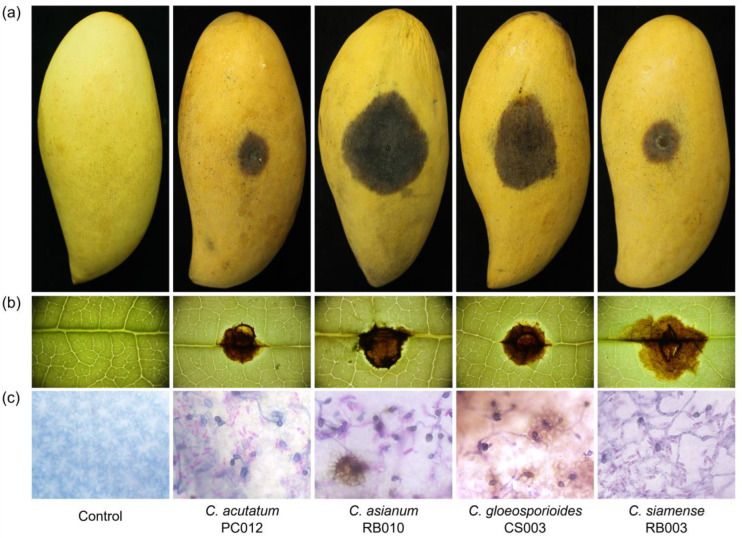
Pathogenicity assay of four different *Colletotrichum* species on NDMST mango; visual anthracnose symptoms developed on fruit (**a**), leaves (**b**) at 5 days after inoculation, and appressoria and spores developed on leaves at 24 h after inoculation (**c**). The test was performed on all isolates; only a representative of each species is shown here.

**Figure 6:**
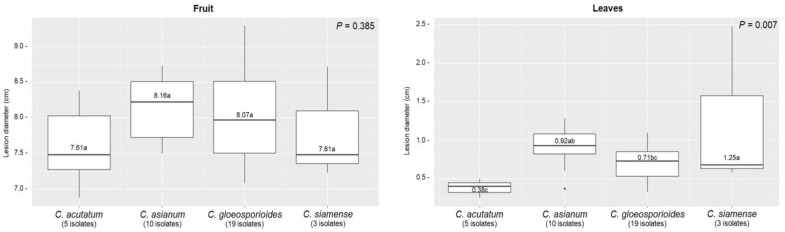
Box plots showing the variation of disease lesion diameter amongst four *Colletotrichum* species associated with mango anthracnose at 5 days after inoculation with a mycelial plug on unwounded NDMST mango fruit and leaves. Vertical lines are median. Means followed by the different common letter are significantly different according to the Tukey-test.

A mycelial plug of a 5-day-old culture was inoculated on unwounded fruit and leaves. Typical anthracnose lesions were observed around the inoculation sites. The anthracnose lesions on fruit enlarged faster than those on leaves on day 5 after inoculation (Table 2 and Figure 5). No lesions were observed on the control fruit or leaves (Figure 5). On inoculated fruit, *C. asianum* produced the largest lesions that differed significantly from other species with the average LD of 8.16 cm, followed by the LD means of *C. gloeosporioides*, *C. siamense*, and *C. acutatum* at 8.07, 7.81, and 7.61 cm, respectively (Table 2 and Figure 6).

Similar results were observed on inoculated leaves; *C. siamense* showed the largest lesions that differed significantly from other species with the average LD of 1.25 cm, whereas the LD means of *C. asianum*, *C. gloeosporioides* and *C. acutatum* were 0.92, 0.71, and 0.38 cm, respectively (Table 2 and Figure 6). After the pathogenicity test, all the *Colletotrichum* spp. were re-isolated from the infected tissues and confirmed by Koch’s postulates to have identical morphological characteristics as the original isolates.

## 3. Discussion

It has been widely accepted that high genetic variation within *Colletotrichum* species complexes exists due to their wide host range and diverse environments [23,24,25,26,27]. Mango anthracnose has been reported to associate with different species of *Colletotrichum*. This study aimed to accurately identify the species of the *Colletotrichum*–mango system in Thailand, using a combination of morphology, multilocus sequence analyses, and Koch’s postulates. A total of 37 *Colletotrichum* species were isolated from mango anthracnose disease from orchards located in central Thailand. When morphological characteristics such as colony color, growth rate, size, and shape of conidia and appressoria were used, 32 isolates were identified as *C. acutatum*, and five isolates were identified as *C. gloeosporioides*. It is generally accepted that morphological characteristics alone are not sufficient for species identification since variation in traits among species can be similar under different environments, therefore the multilocus phylogenetic approach was integrated to aid in taxonomy.

Several DNA markers have been developed to identify *Colletotrichum* isolates. A multilocus sequence analysis using ITS, *CAL*, or *TUB2* identified *C. alienum*, *C. fructicola*, or *C. tropicale* from other *Colletotrichum* species [27]. Similarly, a study of lupin anthracnose by Alkemade et al. [31] showed that 39 out of 50 isolates belonged to *Colletotrichum lupini*. The authors also used the combination of multilocus analysis (ITS, TUB2, GAPDH, and APN/MAT1) and morphological characteristics to support their taxonomic classification of *Colletotrichum* species complex. This study used phenotypic characters, DNA markers of ITS, *ACT*, *CHS-1*, and *TUB2*, and Koch’s postulates to confirm that all 37 *Colletotrichum* isolates were mango anthracnose pathogens. In addition to the four DNA markers used in this study, *GAPDH* has also been used to identify *C. acutatum* and *C. gloeosporioides* complexes by Damm et al. [23] and Weir et al. [27]. In this study, two phylogenetic trees were constructed using two loci (ITS and *TUB2*) and four loci (ITS, *TUB2*, *ACT* and *CHS-1*). The topology of both trees was similar and further identified the *Colletotrichum* species into *C. acutatum*, *C. gloeosporioides*, *C. asianum* and *C. siamense*. These results indicated that using two loci is sufficient to distinguish these four species of *Colletotrichum*. *Colletotrichum gloeosporioides* and *C. acutatum* were previously reported in 1979 and 2019 [32]; however, we have uncovered for the first time that *C. asianum* and *C. siamense* caused anthracnose in mango in Thailand.

*Colletotrichum asianum* has been reported to be a major species causing mango anthracnose in China [33]. It has been the most common endophytic species of mango in northeastern Brazil [34] and in many countries around the world, such as Australia, China, Colombia, Japan, Malaysia, Philippines, Sri Lanka, and Taiwan [11,12,13,18,19,27,35,36]. *Colletotrichum asianum* is also capable of causing disease in avocado (*Persea americana* Mill.) in Australia [19]. In Thailand, *C. asianum* has been reported to cause disease in coffee (*Coffea arabica* L.) [37] but not in mango until this study.

*Colletotrichum siamense* has been reported to be a major species causing mango anthracnose in China [33] and in eastern Australia [38]. It has a wide host range in tropical and subtropical regions and can infect banana (*Musa* spp.), papaya (*Carica papaya* L.), dragon fruit (*Hylocereus* spp.), guava (*Psidium guajava*) and avocado [12,18,19,27,33,39,40,41,42,43,44,45,46]. It was suggested by James et al. [38] that *C. siamense* is likely to be a common and widespread saprophyte or endophyte because the authors were able to isolate it from asymptomatic fruit of other plants, except mango and avocado. In Thailand, similar to *C. asianum*, *C. siamense* has been reported to cause disease in coffee (*Coffea arabica* L.) [37] but not in mango until this study.

Among the four species identified, *C. gloeosporioides* was a major species causing anthracnose in this study. It was found in every orchard that we isolated. The pathogenicity assay showed that all isolates appeared to be more aggressive on fruit than leaves, probably because fruit contains more sugar that serves as a carbon source for fungal growth. *C. asianum* and *C. gloeosporioides* showed a similar degree of aggressiveness on mango fruit and produced larger lesions than *C. siamense* and *C. acutatum*. The color of lesions were also slightly different on inoculated fruit, *C. asianum* produced dark brown spots, while the other three species produced lighter brown spots. The pathogenicity on the leaf showed that *C. siamense* was the most aggressive compared to other species, possibly due to species-specific host responses. Further testing using different cultivars of mango should be employed to verify this hypothesis.

The impact of this study was twofold: firstly, it has proven that a combination of morphology, DNA multilocus sequence analysis, and Koch’s postulates is a robust identification approach to identify species of *Colletotrichum*, and secondly, our approach enables the discovery of previously unreported anthracnose caused by *C. asianum* and *C. siamense*, in Thailand. This discovery raises a concern regarding the cross-infection potential where the two species can infect different hosts. Therefore, accurate diagnosis is a crucial first step for disease control and prevention. It supports the current quarantine regulations as well as establishes strategies for integrated management of anthracnose disease between orchards. It should be noted that fungal pathogens, although in the same genus, will respond to fungicides differently. Further study is essential to determine the fungicide sensitivity of these four *Colletotrichum* species to help implement the fungicide management strategy.

## 4. Materials and Methods

### 4.1. Fungal Isolation

The infected samples of 25 fruit, 10 leaves, and two inflorescences with typical anthracnose symptoms were collected from eight orchards located in central Thailand: Chachoengsao, Phichit, and Ratchaburi, in 2016–2017. The locations of orchards, geographic coordinates, codes of the isolates, and types of infectious tissues collected are provided in Table 1. Thirty-seven isolates of *Colletotrichum* spp. were recovered. The pathogens were isolated and cultured on potato dextrose agar (PDA) using a tissue transplantation technique. To obtain pure isolates, mycelial plugs were sub-cultured on fresh PDA, and incubated at 25 °C under a photoperiod of 12 h light/12 h dark for 5 days [8], followed by single spore isolation on water agar (WA). The single spore from each isolate was transferred to a new PDA plate and incubated under the same condition. Each colony served as a single genetic source for further analysis.

### 4.2. Morphological Characteristics

Each isolate was inoculated with a 6-mm-diameter plug taken from an actively growing edge of a 5-day-old culture on a PDA plate. The culture was incubated under the same condition mentioned above. Fungal growth and colony diameter were recorded daily until there were no changes in diameter (day 3). Fifty conidia were randomly selected for measurement of their length and width at day 5 (conidia were fully matured) under an Olympus CX31 binocular compound microscope at 400× magnification with the Olympus CellSens standard software version 1.16 (Olympus Co., Ltd., Tokyo, Japan). Appressoria were induced using a slide culture technique [47]. Briefly, the isolates were transferred onto 25.4 × 76.2 mm sterile microscope slides (7101 microscope slides, Shandong Harmowell Trade Co., Ltd., Shanghai, China) and covered with 22 × 22 mm coverslips (Menzel Gläser, Thermo Fisher Scientific Co., Ltd., Waltham, MA, USA) incubated in a petri dish at 25 °C until maturation, usually for 4 days. Lengths and widths of 30 appressoria per isolate were measured under a microscope. This experiment was performed in five replicates.

### 4.3. DNA Extraction and Molecular Identification

Genomic DNA was extracted from a 5-day-old fungal colony, according to Pongpisutta et al. [48]. A PCR mixture contained genomic DNA (20 ng), primers (0.48 µM each, Table 3), *Taq* polymerase buffer (1×, Thermo Fisher Scientific, Waltham, MA, USA), MgCl_2_ (2.4 mM), dNTPs (10 µM each), *Taq* polymerase (1 U, Thermo Fisher Scientific, Waltham, MA, USA). PCR was performed in a thermal cycler (Sensoquest GmbH, Göttingen, Germany). The PCR was programmed as follows: 94 °C for 2 min, 35 cycles of denaturation at 94 °C for 30 s, annealing at 55–58 °C (Table 3) for 30 s, and extension at 72 °C for 1 min, with a final extension step of 72 °C for 10 min. The PCR products were visualized on 1.2% agarose gels stained with GelStar^®^ and GeneRuler, and 100 bp Plus DNA Ladder (Thermo Fisher Scientific, Waltham, MA, USA) was used to determine the size of DNA fragments. The PCR products were sequenced by the 1st Base Laboratory Co., Ltd., Seri Kembangan, Malaysia. A basic local alignment search, BLASTn (https://blast.ncbi.nlm.nih.gov/Blast.cgi, accessed on 22 April 2021), was performed to analyze nucleotide sequences in comparison to reference sequences available in the National Center for Biotechnology Information (NCBI) database.

### 4.4. Phylogenetic Analyses

Multiple sequence alignments were performed using ClustalW alignment [52] implemented in MEGA version X [53] and were manually adjusted to allow maximum sequence similarity. Bayesian inference (BI) was used to reconstruct the phylogenetic trees using MrBayes version 3.2.7 [54] implemented in the CIPRES cluster (https://www.phylo.org/portal2/home.action, accessed on 12 December 2021). The nucleotide substitution model was determined by jModelTest v. 2.1.7 [55]. Following Drummond and Rambaut [56], 1,000,000 generations (four chains, four independent runs) were set up, and the analyses were sampled every 1000 generations, with the first 25% of the samples discarded. Maximum likelihood analyses were conducted by the MEGA version X [53] using a TN93+G substitution model based on 1000 bootstrap replicates.

### 4.5. Pathogenicity Test

Harvested mature mango fruit and leaves were washed under running water, immersed in 1.2% sodium hypochlorite solution for 2 min, rinsed twice with sterile distilled water, and allowed to dry under a laminar flow hood. Thirty-seven isolates of *Colletotrichum* were used for the pathogenicity test. Each *Colletotrichum* isolate was inoculated on unwounded fruit and leaves as described in Pongpisutta et al. [8]. Briefly, a mycelial plug from the growing edge of 5-day-old PDA culture was placed onto the fruit and leaf surface of the NDMST cultivar. A control treatment was performed using a non-colonized agar plug. The inoculated samples were placed on trays lined with sterile moist paper towels and kept in sealed plastic bags. Five days after inoculation at room temperature, evaluation of the virulence was performed by measurement of lesion diameter (LD). This experiment was performed in a completely randomized design (CRD) with ten replicates. One-way ANOVA was performed using R software version 3.5.2 [57] with the agricolae package (Statistical procedures for agricultural research) [58]. The means of the LDs were compared by the least significant difference (LSD) test. Additionally, variation of disease lesion diameters on NDMST mango fruit and leaves was compared amongst four *Colletotrichum* species by using box plot analysis which was created in R software likewise.

## 5. Conclusions

A combination of multilocus phylogenetic analysis, phenotypic characters, and Koch’s postulates, provides an effective strategy to overcome the problem of identification and characterization of fungal species. We showed that molecular analysis of at least two loci (ITS and *TUB2*) provides accurate identification of *Colletotrichum* species causing anthracnose disease in mango from central Thailand. This study represents the first report that *C. asianum* and *C. siamense* were found to be causative agents of mango anthracnose in Thailand.

## Figures and Tables

**Table 1:** Collection of the *Colletotrichum* isolates with their geographical information. All isolates were from the NDMST cultivar.

Location of Origin	Geographic Coordinates ^1^	Isolate Code	Source
Plaeng Yao 1, Chachoengsao	13°36′28.6″ N 101°17′53.5″ E	CS001	Fruit
		CS002	Fruit
		CS003	Fruit
Plaeng Yao 2, Chachoengsao	13°36′42.2″ N 101°17′41.6″ E	CS004	Fruit
		CS005	Leaf
		CS006	Leaf
		CS007	Inflorescence
Plaeng Yao 2, Chachoengsao	13°36′50.2″ N 101°17′24.8″ E	CS008	Fruit
		CS009	Fruit
		CS010	Leaf
Sak Lek 1, Phichit	16°28′20.9″ N 100°33′42.4″ E	PC001	Fruit
		PC002	Fruit
		PC003	Fruit
		PC004	Leaf
		PC005	Fruit
		PC006	Leaf
		PC007	Fruit
Sak Lek 2, Phichit	16°28′41.7″ N 100°33′52.4″ E	PC008	Leaf
		PC009	Fruit
		PC010	Leaf
		PC011	Leaf
		PC012	Leaf
Mueang Ratchaburi, Ratchaburi	13°35′50.0″ N 99°49′57.1″ E	RB001	Fruit
		RB002	Leaf
Paktho, Ratchaburi	13°24′47.5″ N 99°45′32.9″ E	RB003	Fruit
		RB004	Inflorescence
		RB005	Fruit
		RB006	Fruit
		RB007	Fruit
		RB008	Fruit
		RB009	Fruit
		RB010	Fruit
		RB011	Fruit
		RB012	Fruit
Bang Phae, Ratchaburi	13°39′50.5″ N 99°57′54.4″ E	RB013	Fruit
		RB014	Fruit
		RB015	Fruit

^1^ Geographic location was detected by GPS status version 8.0.170.

**Table 2:** Lesion sizes on NDMST mango fruit and leaves after inoculation by the 37 isolates of *Colletotrichum* spp.

Isolate Code	Taxon	Lesion Diameter (cm) *	
		Fruit	Leaves
CS001	*C. gloeosporioides*	9.30a	0.33gh
CS002	*C. gloeosporioides*	9.07ab	0.58c–h
CS003	*C. gloeosporioides*	7.97e–j	0.45d–h
CS004	*C. gloeosporioides*	9.00abc	0.75b–h
CS005	*C. gloeosporioides*	8.62b–e	1.12bc
CS006	*C. gloeosporioides*	7.85f–k	1.03b–e
CS007	*C. gloeosporioides*	8.53b–f	0.65b–h
CS008	*C. gloeosporioides*	8.32c–h	0.80b–h
CS009	*C. gloeosporioides*	8.50b–f	0.43e–h
CS010	*C. gloeosporioides*	8.38b–g	0.48c–h
PC001	*C. asianum*	8.52b–f	0.60c–h
PC002	*C. siamense*	8.72a–d	0.58c–h
PC003	*C. asianum*	8.67a–e	0.82b–h
PC004	*C. asianum*	8.48b–f	1.10bcd
PC005	*C. asianum*	8.73a–d	0.93b–g
PC006	*C. acutatum*	6.88m	0.32gh
PC007	*C. acutatum*	8.38b–g	0.25h
PC008	*C. gloeosporioides*	7.47i–m	0.82b–h
PC009	*C. gloeosporioides*	7.53i–m	0.93b–g
PC010	*C. gloeosporioides*	7.18klm	0.35fgh
PC011	*C. acutatum*	8.03d–i	0.45d–h
PC012	*C. acutatum*	7.48i–m	0.40e–h
RB001	*C. asianum*	7.67h–l	0.37fgh
RB002	*C. gloeosporioides*	7.48i–m	0.72b–h
RB003	*C. siamense*	7.48i–m	0.68b–h
RB004	*C. gloeosporioides*	7.63h–l	0.67b–h
RB005	*C. asianum*	7.65h–l	0.82b–h
RB006	*C. siamense*	7.23klm	2.48a
RB007	*C. asianum*	7.50i–m	0.93b–g
RB008	*C. gloeosporioides*	7.73g–l	0.73b–h
RB009	*C. gloeosporioides*	8.40b–g	0.88b–h
RB010	*C. asianum*	8.13d–i	1.28b
RB011	*C. asianum*	7.88f–k	1.28b
RB012	*C. asianum*	8.32c–h	1.03b–e
RB013	*C. gloeosporioides*	7.28j–m	0.80b–h
RB014	*C. acutatum*	7.27j–m	0.50c–h
RB015	*C. gloeosporioides*	7.08lm	1.00b–f

* Values with the same column followed by different common letters mean that they are significantly different based on variance with the least significant difference test at *p* = 0.05.

**Table 3:** Primers used in this study with sequences and sources.

Gene	Primer	Sequence (5′-3′)	Annealing Temperature (°C)	References
Actin	ACT-512F	ATGTGCAAGGCCGGTTTCGC	58	[49]
	ACT-783R	TACGAGTCCTTCTGGCCCAT		
β-tubulin	T1	AACATGCGTGAGATTGTAAGT	55	[50]
	T2	TAGTGACCCTTGGCCCAGTTG		
Chitin synthase 1	CHS-79F	TGGGGCAAGGATGCTTGGAAGAAG	58	[49]
	CHS-345R	TGGAAGAACCATCTGTGAGAGTTG		
ITS region	ITS5	GGAAGTAAAAGTCGTAACAAGG	56	[51]
	ITS4	TCCTCCGCTTATTGATATGC		

## Data Availability

Not applicable.

## Supplementary Materials

The following supporting information can be downloaded at: https://www.mdpi.com/article/10.3390/plants12051130/s1, Table S1: Morphological characteristics of colonies, conidia, and appressoria of 37 mango *Colletotrichum* isolates from central Thailand. Table S2: *Colletotrichum* isolates used in phylogenetic analysis, including all isolates in this study.
