# Supplementary material for: Two Newly Identified Colletotrichum Species Associated with Mango Anthracnose in Central Thailand
Source: Plants (Basel). 2023 Mar 2;12(5):1130. doi: 10.3390/plants12051130 (PMC10004820; doi:10.3390/plants12051130)
Supplement: Supplementary file 1 [file plants-12-01130-s001.zip › plants-2113210-supplementary.pdf]

Table S1. Morphological characteristics of colonies, conidia and appressoria of 37 mango *Colletotrichum* isolates from central Thailand.

Isolate code	Taxon	Growth rate at day 3 (mm)	Colony type	Conidial shape	Conidial size (µm)	Appressorial shape	Appressorial size (µm)
CS001	*C. gloeosporioides*	4.60a-d	Greenish to grey aerial mycelium, reverse pale to dark green. Conidial mass peach	Straight - cylindrical obtuse at the apex one end	2.88-5.71 x 9.45-16.59	Clavate, long clavate to irregular	4.69 - 8.27 x 8.37 - 14.11
CS002	*C. gloeosporioides*	5.37a	Greyish flat mycelium, reverse pale orange to dark grey. Conidia pale salmon to salmon	Straight - cylindrical obtuse at the apex one end	3.29-5.70 x 11.69-16.52	Clavate, long clavate to circular	3.76 - 4.91 x 5.59 - 14.51
CS003	*C. gloeosporioides*	3.60efg	White to pinkish aerial mycelium, reverse yellow to pink. Conidial mass buff	Straight - cylindrical obtuse at the apex one end	3.18-6.49 x 10.48-24.54	Long clavate to irregular	5.24 – 8.24 x 8.31 – 11.84
CS004	*C. gloeosporioides*	5.20ab	White aerial mycelium, reverse dark green. Conidial mass pale salmon	Straight - cylindrical obtuse at the apex one end	2.90-5.59 x 9.35-18.69	Long clavate to irregular	3.00 – 4.75 x 5.88 – 9.71
CS005	*C. gloeosporioides*	4.63a-d	White to olive aerial mycelium, reverse dark green. Conidial mass peach	Straight - cylindrical obtuse at the apex one end	2.90-5.51 x 11.00-17.62	Long clavate to irregular	4.11 – 8.08 x 6.25 – 12.09
CS006	*C. gloeosporioides*	2.13h	White aerial mycelium, reverse dark green. Conidial mass buff to pale salmon	Straight - cylindrical obtuse at the apex one end	2.95-5.50 x 12.05-19.70	Long clavate to irregular	4.25 – 6.93 x 7.55 – 12.02
CS007	*C. gloeosporioides*	5.27a	Greyish white to olive aerial mycelium, reverse dark green. Conidial mass pale salmon	Straight - cylindrical obtuse at the apices	3.79-6.67 x 5.90-18.49	Clavate, long clavate to circular	6.19 – 8.77 x 7.14 – 9.72
CS008	*C. gloeosporioides*	5.03abc	White aerial mycelium, reverse yellowish. Conidial mass buff to pale salmon	Straight - cylindrical obtuse at the apex one end	4.03-6.36 x 13.10-16.77	Clavate, long clavate to irregular	4.46 – 6.98 x 6.25 – 10.75

Table S1. *(Cont.)*

Isolate code	Taxon	Growth rate at day 3 (mm)	Colony type	Conidial shape	Conidial size (µm)	Appressorial shape	Appressorial size (µm)
CS009	*C. gloeosporioides*	4.20c-f	White aerial mycelium, reverse dark green. Conidial mass buff to pale salmon	Straight - cylindrical obtuse at the apex one end	3.30-6.91 x 9.80-26.42	Clavate, long clavate to circular	4.38 – 9.20 x 7.40 – 14.26
CS010	*C. gloeosporioides*	5.43a	White to olive aerial mycelium, reverse dark green. Conidial mass peach	Straight - cylindrical obtuse at the apex one end	3.87-6.68 x 12.49-18.49	Clavate, long clavate to irregular	3.86 – 6.36 x 4.68 – 10.63
PC001	*C. asianum*	3.83d-g	Greenish white to greyish green aerial mycelium, reverse pale yellowish to dark green. Conidial mass peach to salmon orange	Straight - cylindrical obtuse at the apex one end	3.80-7.41 x 12.08-19.14	Clavate, long clavate to irregular	4.13 – 7.08 x 6.99 – 9.39
PC002	*C. siamense*	3.97d-g	White to grey aerial mycelium, reverse pale yellowish to brownish. Conidial mass salmon orange	Straight - cylindrical obtuse at the apex one end	4.12-6.64 x 13.14-19.59	Clavate lobe to irregular	4.19 – 7.32 x 4.60 – 9.66
PC003	*C. asianum*	3.27fg	Greenish white to greyish green aerial mycelium, reverse pale yellowish to dark green. Conidial mass peach to salmon orange	Short cylindrical obtuse at the apex one end	2.95-6.47 x 10.13-16.99	Clavate, long clavate to irregular	3.87 – 7.67 x 5.11 – 11.13
PC004	*C. asianum*	3.93d-g	Greenish white to greyish green aerial mycelium, reverse pale yellowish to dark green. Conidial mass peach to salmon orange	Straight - cylindrical obtuse at the apex one end	4.14-6.22 x 13.81-19.27	Clavate, long clavate to irregular	5.18 – 11.93 x 5.94 – 15.13

Table S1. *(Cont.)*

Isolate code	Taxon	Growth rate at day 3 (mm)	Colony type	Conidial shape	Conidial size (µm)	Appressorial shape	Appressorial size (µm)
PC005	*C. asianum*	3.37efg	Greenish white to greyish green aerial mycelium, reverse dark green. Conidial mass peach to salmon orange	Straight - cylindrical obtuse at the apex one end	4.16-7.12 x 9.87-19.06	Long clavate to irregular	6.03 – 9.26 x 8.64 – 12.03
PC006	*C. acutatum*	3.53efg	White to grey aerial mycelium, reverse pale ochreous. Conidial mass bright orange	Clavate obtuse at the apex one end	3.45-6.15 x 11.80-18.64	Clavate lobe to irregular	4.28 – 7.92 x 6.06 – 12.38
PC007	*C. acutatum*	3.70d-g	White to grey aerial mycelium, reverse pale ochreous. Conidial mass bright orange	Clavate obtuse at the apex one end	3.39-5.71 x 10.22-19.66	Clavate lobe to irregular	2.30 – 5.9 x 4.42 – 6.63
PC008	*C. gloeosporioides*	3.33efg	White to grey aerial mycelium, reverse pale ochreous. Conidial mass bright orange to salmon	Straight - cylindrical obtuse at the apex one end	3.31-8.94 x 9.98-20.09	Clavate, long clavate to irregular	3.80 – 7.12 x 6.28 – 12.06
PC009	*C. gloeosporioides*	3.87d-g	White to grey aerial mycelium, reverse pale ochreous. Conidial mass bright orange to salmon	Straight - cylindrical obtuse at the apex one end	3.89-6.71 x 12.12-18.73	Clavate, long clavate to irregular	4.25 – 8.78 x 4.11 – 7.55
PC010	*C. gloeosporioides*	4.10c-g	White to grey aerial mycelium, reverse pale ochreous. Conidial mass bright orange to salmon	Straight - cylindrical obtuse at the apex one end	3.21-7.02 x 10.23-21.79	Clavate, long clavate to irregular	5.29 – 8.21 x 5.65 – 11.04
PC011	*C. acutatum*	3.77d-g	White to grey aerial mycelium, reverse pale ochreous. Conidial mass bright orange	Clavate obtuse at the apex one end	3.47-6.57 x 10.63-22.79	Clavate lobe to irregular	4.78 – 7.78 x 7.85 – 11.70
PC012	*C. acutatum*	3.33efg	White to grey aerial mycelium, reverse pale ochreous. Conidial mass bright orange	Clavate obtuse at the apex one end	3.31-6.38 x 12.12-20.17	Clavate lobe to irregular	3.56 – 5.62 x 4.75 – 12.61

Table S1. *(Cont.)*

Isolate code	Taxon	Growth rate at day 3 (mm)	Colony type	Conidial shape	Conidial size (µm)	Appressorial shape	Appressorial size (µm)
RB001	*C. asianum*	3.27fg	Greenish white and becoming greyish green aerial mycelium, reverse pale yellowish. Conidial mass peach to salmon orange	Straight - cylindrical obtuse at the apex one end	4.25-6.41 x 12.49-17.00	Clavate to circular	3.51 – 9.10 x 5.59 – 19.60
RB002	*C. gloeosporioides*	3.17g	White to grey aerial mycelium, reverse pale ochreous. Conidial mass bright orange	Straight - cylindrical obtuse at the apex one end	3.84-6.43 x 11.06-17.75	Clavate to circular	4.35 – 6.58 x 5.33 – 8.59
RB003	*C. siamense*	4.20c-f	White aerial mycelium, reverse pale yellowish. Conidial mass bright orange to salmon orange	Clavate - cylindrical obtuse at the apex one end	4.22-7.05 x 10.68-15.05	Clavate lobe to irregular	5.28 – 6.85 x 6.13 – 9.46
RB004	*C. gloeosporioides*	4.30b-e	White aerial mycelium, reverse pale yellowish. Conidial mass bright orange to buff	Straight - cylindrical obtuse at the apex one end	4.39-6.10 x 12.60-16.75	Clavate to circular	4.03 – 5.92 x 6.59 – 9.82
RB005	*C. asianum*	3.37efg	Greenish white and becoming greyish green aerial mycelium, reverse pale yellowish. Conidial mass peach to salmon orange	Straight - cylindrical obtuse at the apex one end	2.63-6.68 x 9.03-21.27	Long clavate irregular to lobe	4.42 – 6.31 x 6.98 – 11.78
RB006	*C. siamense*	4.03d-g	White aerial mycelium, reverse pale yellowish. Conidial mass bright orange to salmon orange	Short cylindrical obtuse at the apex one end	4.78-7.50 x 7.35-14.29	Clavate lobe to irregular	4.82 – 7.41 x 5.85 – 12.67
RB007	*C. asianum*	3.93d-g	Greenish white to greyish green aerial mycelium, reverse pale yellowish to dark green. Conidial mass peach to salmon orange	Straight - cylindrical obtuse at the apex one end	4.29-6.16 x 12.47-18.48	Clavate, long clavate to irregular	4.37 – 6.28 x 5.07 – 8.55

Table S1. *(Cont.)*

Isolate code	Taxon	Growth rate at day 3 (mm)	Colony type	Conidial shape	Conidial size (µm)	Appressorial shape	Appressorial size (µm)
RB008	*C. gloeosporioides*	4.13c-g	White aerial mycelium, reverse pale yellowish. Conidial mass peach to buff	Clavate - cylindrical obtuse at the apex one end	3.99-8.38 x 12.62-17.53	Clavate, long clavate to irregular	3.97 – 6.65 x 5.91 – 12.00
RB009	*C. gloeosporioides*	4.70a-d	White aerial mycelium, reverse pale yellowish. Conidial mass salmon	Straight - cylindrical obtuse at the apex one end	4.98-11.14 x 13.29-20.64	Long clavate irregular to lobe	4.53 – 8.13 x 7.67 – 12.18
RB010	*C. asianum*	3.27fg	Greenish white to greyish green aerial mycelium, reverse pale yellowish to dark green. Conidial mass peach to salmon orange	Straight - cylindrical obtuse at the apex one end	3.95-6.46 x 12.55-17.83	Clavate, long clavate to irregular	4.10 – 7.72 x 7.58 – 13.79
RB011	*C. asianum*	3.37efg	Greenish white to greyish green aerial mycelium, reverse pale yellowish to dark green. Conidial mass peach to salmon orange	Straight - cylindrical obtuse at the apex one end	4.53-6.65 x 11.51-19.58	Clavate, long clavate to irregular	4.82 – 8.76 x 6.10 – 9.46
RB012	*C. asianum*	3.53efg	Greenish white to greyish green aerial mycelium, reverse pale yellowish to dark green. Conidial mass peach to salmon orange	Straight - cylindrical obtuse at the apex one end	3.53-8.58 x 12.69-26.89	Clavate, long clavate to irregular	3.80 – 6.85 x 4.96 – 10.42
RB013	*C. gloeosporioides*	3.97d-g	Greenish white to greyish green aerial mycelium, reverse pale yellowish to dark green. Conidial mass pale to salmon orange	Straight - cylindrical obtuse at the apex one end	2.94-6.14 x 13.02-20.29	Clavate to circular	4.14 – 8.05 x 7.20 – 10.56
RB014	*C. acutatum*	3.47efg	White to grey aerial mycelium, reverse pale ochreous. Conidial mass bright orange	Clavate obtuse at the apex one end	3.29-6.11 x 9.91-17.55	Clavate, long clavate to irregular	4.36 – 7.41 x 6.80 – 12.69

Table S1. *(Cont.)*

Isolate code	Taxon	Growth rate at day 3 (mm)	Colony type	Conidial shape	Conidial size (µm)	Appressorial shape	Appressorial size (µm)
RB015	*C. gloeosporioides*	4.10c-g	Greenish white to greyish green aerial mycelium, reverse pale yellowish to dark green. Conidial mass peach to buff	Straight - cylindrical obtuse at the apex one end	4.08-6.54 x 11.90-19.65	Clavate to irregular	4.93 – 6.91 x 7.23 – 16.77

Table S2. *Colletotrichum* isolates used in phylogenetic analysis, including all isolates in this study.

Species	Accession No.[1]	Host/Substrate	Country	GeneBank No.			
				ACT	*CHS-1*	*ITS*	*TUB2*
C. acerbum	CBS128530, ICMP12921, PRJ1199.3*	*Malus domestica*	New Zealand	JQ949780	JQ949120	JQ948459	JQ950110
C. acutatum	IMI223120, CPC18870	*Anemone* sp.	Australia	JQ949674	JQ949014	JQ948353	JQ950004
	CBS144.29	*Capsicum annuum*	Sri Lanka	JQ949722	JQ949062	JQ948401	JQ950052
	CBS112996, ATCC56816, STE-U5292*	*Carica papaya*	Australia	JQ005839	JQ005797	JQ005776	JQ005860
	IMI216370, CPC18869	*Coffea arabica*	Tanzania	JQ949719	JQ949059	JQ948398	JQ950049
	CBS979.69	*Coffea arabica*	Kenya	JQ949721	JQ949061	JQ948400	JQ950051
	PC006	*Mangifera indica*	Thailand	LC660217	LC660197	MK212357	LC635542
	PC007	*Mangifera indica*	Thailand	LC660218	LC660198	MK212356	LC635543
	PC011	*Mangifera indica*	Thailand	LC618307	LC618325	MK215690	LC635544
	PC012	*Mangifera indica*	Thailand	LC660221	LC660201	MK215688	LC635545
	RB014	*Mangifera indica*	Thailand	LC618308	LC618324	MK215706	LC635541
C. aenigma	ICMP18608*	*Persea americana*	Israel	JX009443	JX009774	JX010244	JX010389
C. aeschynomenes	ICMP17673*, ATCC201874	*Aeschynomene virginica*	USA	JX009483	JX009799	JX010176	JX010392
C. alienum	ICMP12071*	*Malus domestica*	New Zealand	JX009572	JX009882	JX010251	JX010411
C. annellatum	CBS129826, CH1*	*Hevea indica*	Columbia	JQ005570	JQ005396	JQ005222	JQ005656
C. aotearoa	ICMP18535	*Dacrycarpus dacrydioides*	New Zealand	JX009545	JX009766	JX010201	JX010423
C. asianum	IMI313839, ICMP18696	*Mangifera indica*	Australia	JX009576	JX009753	JX010192	JX010384
	NN19	*Mangifera indica*	China	MF039745	MF039774	MF039832	MF039803
	WM52	*Mangifera indica*	China	MF039746	MF039775	MF039833	MF039804
	NN8	*Mangifera indica*	China	MF039754	MF039783	MF039841	MF039812
	ICMP18580*, CBS130418	*Coffea arabica*	Thailand	JX009584	JX009867	FJ972612	JX010406
	PC001	*Mangifera indica*	Thailand	LC618298	LC618315	MK212353	LC635534
	PC003	*Mangifera indica*	Thailand	LC618299	LC618316	MK212354	LC635535
	PC004	*Mangifera indica*	Thailand	LC618300	LC618317	MK215703	LC635536
	PC005	*Mangifera indica*	Thailand	LC618301	LC618318	MK215686	LC635537
	RB001	*Mangifera indica*	Thailand	LC616858	LC618309	MK215695	LC635528
	RB005	*Mangifera indica*	Thailand	LC616859	LC618310	MK215689	LC635529
	RB007	*Mangifera indica*	Thailand	LC618294	LC618311	MK215692	LC635530
	RB010	*Mangifera indica*	Thailand	LC618295	LC618312	MK215694	LC635531
	RB011	*Mangifera indica*	Thailand	LC618296	LC618313	MK215701	LC635532
	RB012	*Mangifera indica*	Thailand	LC618297	LC618314	MK215700	LC635533
C. australe	CBS116478, HKUCC2616*	*Trachycarpus sfortunei*	South Africa	JQ949776	JQ949116	JQ948455	JQ950106
C. beeveri	CBS128527, ICMP18594*	*Brachyglottis repanda*	New Zealand	JQ005519	JQ005345	JQ005171	JQ005605
C. boninense	CBS123756, MAFF306094	*Crinum asiaticum* var. *sinicum*	Japan	JQ005502	JQ005328	JQ005154	JQ005589
C. brassicicola	CBS101059, LYN16331*	*Brassica oleracea* var. *gemmifera*	New Zealand	JQ005520	JQ005346	JQ005172	JQ005606
C. brisbanense	CBS292.67, DPI11711*	*Capsicum annuum*	Australia	JQ949612	JQ948952	JQ948291	JQ949942
C. colombiense	CBS129817, G1	*Passiflora edulis*	Columbia	JQ005521	JQ005347	JQ005173	JQ005607
C. constrictum	CBS128504, ICMP12941*	*Citrus limon*	New Zealand	JQ005586	JQ005412	JQ005238	JQ005672
C. fructicola	ICMP18581*, CBS130416	*Coffea arabica*	Thailand	FJ907426	JX009866	JX010165	JX010405
C. ignotum	CBS125397*, ICMP1846	*Tetragastris panamensis*	Panama	JX009581	JX009874	JX010173	JX010409
C. gloeosporioides	IMI356878*, ICMP17821, CBS112999	*Citrus sinensis*	Italy	JX009531	JX009818	JX010152	JX010445

Table S2. *(Cont.)*.

Species	Accession No.[1]	Host/Substrate	Country	GeneBank No.			
				ACT	*CHS-1*	*ITS*	*TUB2*
	CS001	*Mangifera indica*	Thailand	LC660202	LC660182	MK128499	LC628900
	CS002	*Mangifera indica*	Thailand	LC660203	LC660183	MK212348	LC628901
	CS003	*Mangifera indica*	Thailand	LC660204	LC660184	MK212349	LC628902
	CS004	*Mangifera indica*	Thailand	LC660205	LC660185	MK212350	LC628903
	CS005	*Mangifera indica*	Thailand	LC660206	LC660186	MK212351	LC628904
	CS006	*Mangifera indica*	Thailand	LC660207	LC660187	MK215702	LC628905
	CS007	*Mangifera indica*	Thailand	LC660208	LC660188	MK215691	LC628906
	CS008	*Mangifera indica*	Thailand	LC618302	LC618321	MK212352	LC628907
	CS009	*Mangifera indica*	Thailand	LC660209	LC660189	MK215693	LC628908
	CS010	*Mangifera indica*	Thailand	LC660210	LC660190	MK215704	LC628909
	PC008	*Mangifera indica*	Thailand	LC660219	LC660199	MK215687	LC635525
	PC009	*Mangifera indica*	Thailand	LC618304	LC618323	MK215697	LC635526
	PC010	*Mangifera indica*	Thailand	LC660220	LC660200	MK215696	LC635527
	RB002	*Mangifera indica*	Thailand	LC660211	LC660191	MK212942	LC635519
	RB004	*Mangifera indica*	Thailand	LC618303	LC618322	MK215698	LC635520
	RB008	*Mangifera indica*	Thailand	LC660213	LC660193	MK212948	LC635521
	RB009	*Mangifera indica*	Thailand	LC660214	LC660194	MK212947	LC635522
	RB013	*Mangifera indica*	Thailand	LC660215	LC660195	MK215707	LC635523
	RB015	*Mangifera indica*	Thailand	LC660216	LC660196	MK215705	LC635524
C. horii	NBRC7478*, ICMP10492	*Diospyros kaki*	Japan	JX009438	JX009752	GQ329690	JX010450
C. karstii	CBS127596, BRIP28443A	*Mangifera indica*	Australia	JQJ005551	JQ005377	JQ005203	JQ005637
C. musae	CBS116870*, ICMP19119	*Musa* sp.	USA	JX009433	JX009896	JX010146	HQ596280
C. nymphaeae	CBS129926, CPC18719	litter	Thailand	JQ949537	JQ948877	JQ948216	JQ949867
C. orchidophilum	CBS632.80*	*Dendrobium* sp.	USA	JQ949472	JQ948812	JQ948151	JQ949802
C. petchii	CBS378.94*	*Dracaena marginata*	Italy	JQ005571	JQ005397	JQ005223	JQ005657
C. psidii	CBS145.29*, ICMP19120	*Psidium* sp.	Italy	JX009515	JX009901	JX010219	JX010443
C. queenslandicum	ICMP1778*	*Carica papaya*	Australia	JX009447	JX009899	JX010276	JX010414
C. scovillei	CBS126529, PD94/921-3, BBA70349*	*Capsicum* sp.	Indonesia	JQ949588	JQ948928	JQ948267	JQ949918
C. siamense	ICMP12567	*Persea americana*	Australia	JX009541	JX009761	JX010250	JX010387
	DAR76934, ICMP18574	*Pistacia vera*	Australia	JX009535	JX009798	JX010270	JX010391
	ICMP18121	*Dioscorea rotundata*	Nigeria	JX009460	JX009845	JX010245	JX010402
	ICMP18578*, CBS130417	*Coffea arabica*	Thailand	FJ907423	JX009865	JX010171	JX010404
	ICMP17795	*Malus domestica*	USA	JX009506	JX009805	JX010162	JX010393
	PC002	*Mangifera indica*	Thailand	LC618306	LC618320	MK212355	LC635540
	RB003	*Mangifera indica*	Thailand	LC618305	LC618319	MK212943	LC635538
	RB006	*Mangifera indica*	Thailand	LC660212	LC660192	MK215699	LC635539
C. simmondsii	CBS122122, BRIP28519*	*Carica papaya*	Australia	JQ949597	JQ948937	JQ948276	JQ949927
C. theobromicola	CBS124945*, ICMP18649	*Theobroma cacao*	Panama	JX009444	JX009869	JX010294	JX010447
C. torulosum	CBS128544, ICMP18586*	*Solanum melongena*	New Zealand	JQ005512	JQ005338	JQ005164	JQ005598
C. tropicale	MAFF239933, ICMP18672	*Litchi chinensis*	Japan	JX009480	JX009826	JX010275	JX010396
Leptosphaeria veronicae	CBS:145.84	*Veronica chamaedryoides*	Netherlands	JF740142	JF740178	JF740254	JF740160

[1]BRIP: Plant Pathology Herbarium, Department of Employment, Economic, Development and Innovation, Queensland, Australia; CBS: Culture collection of the Centraalbureau voor Schimmel cultures, Fungal Biodiversity Centre, Utrecht, The Netherlands; HKUCC: The University of Hong Kong Culture Collection, Hong Kong, China; ICMP: International Collection of Microorganisms from Plants, Auckland, New Zealand; IMI: Culture collection of CABI Europe UK Centre, Egham, UK; MAFF: MAFF GeneBank Project, Ministry of Agriculture, Forestry and Fisheries, Tsukuba, Japan; NBRC = Biological Resource Center, National Institute of Technology and Evaluation (Japan); PD: Plantenziektenkundige Dienst Wageningen, the Netherlands; and STE-U: Culture collection of the Department of Plant Pathology, University of Stellenbosch, South Africa.

* ex-holotype or ex-epitype cultures
